# Reprogramming tumor-associated macrophages using STING or TLR agonists: a promising strategy to enhance immunotherapy in hormone-dependent cancers

**DOI:** 10.1136/jitc-2024-010950

**Published:** 2025-05-15

**Authors:** Victor Sacristan Santos, Alba Pensado-López, Rosario García-Campelo, Silvia Antolin Novoa, Rosa Señaris Rodriguez, Fernando Torres Andón

**Affiliations:** 1 Medical Oncology Department, INIBIC, A Coruna, Galicia, Spain; 2 Complexo Hospitalario Universitario A Coruña, A Coruna, Galicia, Spain; 3 Department of Physiology, Centro de Investigación en Medicina Molecular y Enfermedades Crónicas (CiMUS), Universidade de Santiago de Compostela - Campus de Santiago, Santiago de Compostela, Spain

**Keywords:** Macrophage, Toll-like receptor - TLR, Nanoparticle, Solid tumor, Immunotherapy

## Abstract

Hormone-dependent cancers, like breast and prostate cancers, represent a unique challenge in oncology due to their complex interplay between hormone signaling, immune evasion, and therapeutic resistance. While endocrine therapies effectively target hormone signaling to initially control disease, resistance mechanisms frequently emerge, leading to cancer progression and limited survival. These solid tumors further complicate treatment by establishing an immunosuppressive tumor microenvironment (TME), presenting variable numbers of immune cells depending on cancer type and stage, which hinders the efficacy of immune checkpoint inhibitors. In this TME, tumor-associated macrophages (TAMs) are the major cellular source of immunosuppression, supporting tumor growth. The ability of TAMs to hamper the effectiveness of endocrine therapy is becoming increasingly recognized. Reprogramming TAMs within solid tumors can restore their natural ability to fight cancer and also enhance antitumoral efficacy. In this line of research, Al-Janabi *et al* have recently developed lipid nanoparticles decorated with antibodies that bind to the folate receptor-beta overexpressed in perivascular TAMs and loaded with a STING agonist (cGAMP) for the reprogramming of these TAMs. In preclinical murine models of prostate cancer, this therapeutic approach demonstrated significant synergistic activity with androgen deprivation therapy. This work provides an excellent example of TAM reprogramming combined with endocrine therapy for the treatment of hormone-dependent cancers.

 Hormone-dependent cancers encompass a significant subset of tumors, with breast cancer (BC) being the most prevalent in women and prostate cancer (PC) in men. Other common hormone-dependent cancers in women include ovarian and endometrial cancer. Sex steroid hormones, such as estrogen and androgen, stimulate the progression and malignancy of cancer cells in luminal breast and prostate tumors. These hormones act through dedicated receptors, primarily expressed in the nucleus, that are overexpressed in cancer cells. Thus, a broad spectrum of therapeutic approaches, known as endocrine therapies, has been developed to block or inhibit the estrogen receptors (ER) in BC or the androgen receptors (AR) in PC, or other mechanisms along their respective hormone signaling pathways. Current treatments in clinical oncology involve endocrine therapies, but also surgery and cytotoxic drugs or cell cycle inhibitors. With the combination of these therapeutic approaches, median survival rates of approximately 5 years can be achieved in metastatic hormone-dependent tumors, with complete response rates of ~40% in localized BC and ~65% in localized PC.^1 2^ In PC, androgen deprivation therapy (ADT) is the foundation of the most effective treatments. Although initially effective, resistance frequently develops, leading to castration-resistant prostate cancer, often reducing survival to less than 30 months.^1^ In hormone-positive BC (estrogen and/or progesterone), endocrine therapies are initially effective in ~75% of cases, but resistance still appears in about~50% of patients.^2^ Mechanisms of resistance to endocrine therapy in BC and PC include: receptor alterations (mutations, splice variants or amplifications), reliance on non-hormone-mediated pathways, dysregulation of hormone signaling pathways, and also the immunosuppressive tumor microenvironment (TME).^1 2^ Unfortunately, the use of immune checkpoint inhibitors (ICIs) shows limited benefits in patients with PC or BC. Therefore, endocrine therapies initially show promise, but the development of resistance continues to hinder long-term success in many patients. Thus, our understanding of hormone-dependent cancers must be improved.

Substantial evidence in PC and BC points towards the TME as a major driver of resistance. Hormone-dependent cancers are often considered immune “cold” tumors, with variable numbers of immune cells depending on cancer type and stage. These tumors are often characterized by low levels of immune cell infiltration, PD-L1 expression, and somatic mutations, compared with other tumor types like triple-negative breast cancer.^2^ Moreover, several investigations have found an increased frequency of immune effector cells in response to hormone-deprivation therapy, although with negative outcomes, characterized by impairment of T cell cytotoxic functions and an increase in immunosuppressive cells in the TME, such as tumor-associated macrophages (TAMs), myeloid-derived suppressor cells, and regulatory T cells.^1 2^ TAMs are a major cellular component in many solid tumors. These immunosuppressive cells interfere with a wide range of antitumor treatments, hindering the efficacy of conventional therapies, like chemotherapy and radiotherapy, as well as newer approaches such as anti-angiogenic drugs, targeted therapies (TTs) and ICIs.^3^ The immunosuppressive nature of TAMs and the extracellular matrix (ECM) of solid tumors also limits the use of CAR-T cell therapy. More recently, a few studies revealed that TAMs contribute to resistance to endocrine therapies and also limit the efficacy of chemotherapy and ICIs in these tumors.^3^ This evidence highlights the need for strategies to reprogram the TME, particularly TAMs, and to enhance antitumor immunity, ultimately improving responses to endocrine and immunotherapies.

Targeting the monocyte-macrophage system for treating hormone-dependent diseases was already proposed three decades ago, yet progress in this area has been limited. A recent example by Al-Janabi *et al,*^4^ highlights the potential of TAM-targeting and reprogramming for the treatment of hormone-dependent tumors. They developed lipid nanoparticles (NPs) decorated with antibodies that bind to the folate receptor β (FRβ) overexpressed in perivascular TAMs, and loaded with a STING agonist (cGAMP) to reprogram these cells towards M1-like antitumor macrophages. The stimulator of interferon genes (STING) pathway has been described as a promising immunotherapeutic target to trigger innate and adaptive immune activation through the production of type I interferon (IFN-I), with dendritic cells and macrophages as key cellular mediators.^5^ Similar activity has also been found with the use of endosomal TLR (toll-like receptor) agonists.^6^ Thus, several researchers are exploring the use of STING (cGAS/stimulator of interferon genes) or TLR (toll-like receptor) agonists, with or without NPs, for reprogramming TAMs in a variety of solid tumors.^5 6^ Although STING and TLR agonists are natural triggers of innate and adaptive immune responses, applying them for cancer immunotherapy is not an easy task. Key challenges include the route of administration (local or systemic), pharmaceutical formulation, and careful dose control to minimize side effects (ie, immunotoxicity). While STING and TLR agonists have potent immunostimulatory effects on immune cells, particular macrophages, their activity is not sustained.^5 6^ Achieving long-lasting reprogramming of TAMs and the TME is difficult and likely requires continuous administration, combination therapies, and even pharmaceutical formulations for controlled release. For example, we recently reported the superior antitumor efficacy of combining poly(I:C) (TLR3 agonist) with resiquimod (R848, TLR7/8 agonist), compared to imiquimod (TLR7 agonist), or monotherapy with either TLR agonist.^6^ This synergistic effect was mediated by activation of the STAT1 pathway. Furthermore, to improve their intravenously delivery, polymeric nanocapsules were developed, loaded with poly(I:C) and R848, and coated with mannose-functionalized hyaluronic acid to target the CD206 receptor overexpressed on TAMs.^7^ In contrast, Al-Janabi *et al*^4^ employed a novel approach for targeting TAMs in orthotopic murine models of PC. This involved anchoring antibodies against the FRβ, which is overexpressed in perivascular TAMs, on the lipid NPs. It is important to acknowledge that these active targeting approaches, relying on the overexpression of mannose or folate receptors by TAMs, have limited cellular selectivity with implications for off-target effects and safety issues. These receptors are also present on monocytes/macrophages in other anatomical locations (eg, Kupffer cells in the liver), or even on the surface of other cell types and could manifest as autoimmune toxicity. Recently, Guimarães *et al* demonstrated that FRβ-expressing macrophages correlate with poor clinical outcomes in ovarian and triple-negative breast cancers, and they also found a specific macrophage population co-expressing FRβ and PD-L2.^8^ These recent findings suggest the use of FRβ-targeting NPs for other tumor types, and also suggest the addition of several ligands (ie, anti-PD-L2) to improve the selectivity of the NPs towards the right population of TAMs. Other researchers have also demonstrated the accumulation of FRβ-targeting lipid-NPs in other tumor types, such as lung cancer.^9^ In addition to the active targeting, the design of new NPs to target TAMs must also consider the passive targeting of NPs, which relies on the enhanced permeability and retention phenomenon and might be different for different types of solid tumors. This effect is caused by disorganized blood and lymphatic vessels in the tumor, which allows NPs to accumulate in the TME. However, most NPs struggle to penetrate the tumor core due to the dense ECM and high cellular density. To overcome this limitation, Al-Janabi *et al*^4^ developed a strategy to actively target perivascular TAMs, which reside on the surface of the blood vessels within the TME, making them ideally positioned for NP uptake. Once reprogrammed, these TAMs can initiate antitumor innate and adaptive immune responses against the cancer cells.

Moreover, beyond local antitumor activity, the systemic action of STING or TLR agonists is crucial for effective cancer therapy. While Al-Janabi *et al* did not specifically investigate the antimetastatic potential of their TAM-targeted therapy, other studies have demonstrated the systemic effects of STING or TLR agonists.^5 6^ Of note, the intratumoral administration of poly(I:C) and R848 induced long-term activation of innate and adaptive immune responses (CD4 and CD8 T cells), leading to systemic antitumor activity, elimination of metastatic cancer cells, and rejection of tumor rechallenge. Other groups achieved similar results with other TLR agonists (mainly R848 or CpG, agonist of TLR9) or STING agonists (mainly cGAMP),^5 6^ often testing the combination of these drugs with ICIs to enhance T cell response; with radiotherapy or immunogenic cell death inducers to kill cancer cells and enhance antitumor immunity (ie, releasing damage-associated molecular patterns and tumor antigens). Only a few investigations tried the combination of STING or TLR agonists with TTs for well-known mutations, such as EGFR (eg, erlotinib); or with CAR-T cells to improve their ability to fight solid tumors. Now, Al-Janabi *et al*^4^ demonstrated the synergistic antitumor activity of combining STING-loaded NPs with ADT in preclinical murine models of PC. Similarly, Zhang *et al* showed that combining STING agonists with AKT1 inhibitors in endocrine-resistant ER+HER2 BC restored innate and adaptive immune signaling and suppressed tumor growth.^10^ These findings highlight the potential of combining endocrine therapies, intended to prevent tumor growth, with TAM reprogramming, aimed to unleash innate and adaptive immune responses in the TME, as well as recover the natural ability of macrophages to phagocyte and kill the cancer cells (figure 1), altogether enhancing antitumor immunity and overcoming therapeutic resistance.

**Figure 1:**
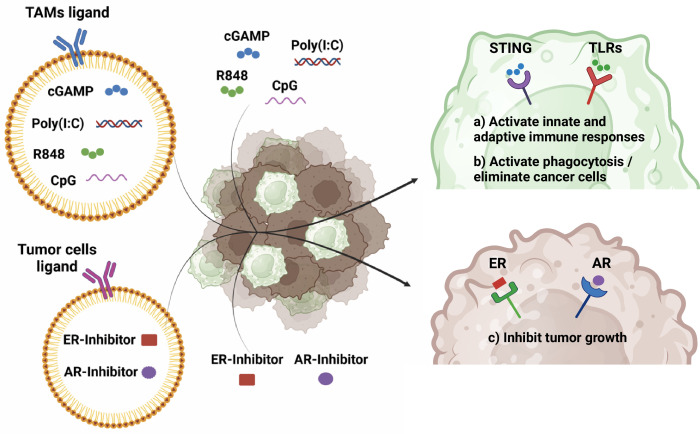
Schematic diagram of combination therapies involving endocrine therapies and TAM reprogramming with STING or TLR agonists, with or without nanoparticles (NPs). The development of NPs to improve the ability of STING or TLR agonists to reach their endosomal receptors and reprogram TAMs, combined with NPs delivering endocrine therapies towards the cancer cells, has the potential to improve antitumoral efficacy through: (**a**) activating innate and adaptive immune responses; (**b**) inducing phagocytosis and/or eliminating cancer cells by macrophages; (**c**) and inhibiting tumor growth by blocking endocrine pathways. Created in BioRender.com. AR, androgen receptor; ER, estrogen receptor; STING, stimulator of interferon gene; TAM, tumor-associated macrophage; TLR, toll-like receptor.

As a whole, although endocrine therapies initially show promise, resistance limits their long-term effectiveness. In hormone-dependent tumors, the immunosuppressive TME plays a key role in this resistance, highlighting the need for strategies like TAM-targeted approaches to reprogram the TME and enhance antitumor immunity, reducing reliance on more toxic treatments like radiotherapy or chemotherapy, thereby improving patients’ quality of life. In particular, the combination of TAM reprogramming with endocrine therapies (figure 1) offers a promising avenue for overcoming resistance and ultimately improving patient outcomes in hormone-dependent cancers.
